# The Influence of Family History of Type 2 Diabetes on Metabolism during Submaximal Aerobic Exercise and in the Recovery Period in Postmenopausal Women

**DOI:** 10.3390/nu14214638

**Published:** 2022-11-03

**Authors:** Jean-Christophe Lagacé, Jasmine Paquin, Renaud Tremblay, Philippe St-Martin, Daniel Tessier, Mélanie Plourde, Eléonor Riesco, Isabelle J. Dionne

**Affiliations:** 1 Research Centre on Aging, Affiliated with CIUSSS de l’Estrie-CHUS, 1036, Rue Belvédère Sud, Sherbrooke, QC J1H 4C4, Canada; 2 Faculty of Physical Activity Sciences, University of Sherbrooke, 2500, Boul. De l’Université, Sherbrooke, QC J1K 2R1, Canada; 3 Faculty of Medicine and Health Sciences, University of Sherbrooke, 2500, Boul. De l’Université, Sherbrooke, QC J1K 2R1, Canada

**Keywords:** family history of type 2 diabetes, postmenopausal women, exercise, substrate oxidation, metabolism

## Abstract

Aging and family history of type 2 diabetes (T2D) are known risk factors of T2D. Younger first-degree relatives (FDR) of T2D patients have shown early metabolic alterations, which could limit exercise’s ability to prevent T2D. Thus, the objective was to determine whether exercise metabolism was altered during submaximal exercise in FDR postmenopausal women. Nineteen inactive postmenopausal women (control: 10, FDR: 9) aged 60 to 75 years old underwent an incremental test on a cycle ergometer with intensity ranging from 40 to 70% of peak power output. Participants consumed 50 mg of ^13^C-palmitate 2 h before the test. At the end of each stage, glucose, lactate, glycerol, non-esterified fatty acids and ^13^C-palmitate were measured in plasma, and ^13^CO_2_ was measured in breath samples. Gas exchanges and heart rate were both monitored continuously. There were no between-group differences in substrate oxidation, plasma substrate concentrations or ^13^C recovered in plasma or breath. Interestingly, despite exercising at a similar relative intensity to control, FDR were consistently at a lower percentage of heart rate reserve. Overall, substrate plasma concentration and oxidation are not affected by family history of T2D in postmenopausal women and therefore not a participating mechanism in the altered response to exercise previously reported. More studies are required to better understand the mechanisms involved in this response.

## 1. Introduction

It is well recognized that aging individuals have a greater prevalence of type 2 diabetes (T2D) than younger individuals [1]. With the menopausal transition, aging women go through metabolic and hormonal changes which affect body weight, body composition and adipose tissue distribution which, in turn, can lead to an increased risk of T2D [2]. In parallel to older age and menopausal transition, having a family history of T2D is also recognized as a risk factor for T2D [3]. In first-degree relatives (FDR) of individuals with T2D, early metabolic and/or anthropometric alterations have been identified as possible contributors to this risk. Evidence show that FDR are likely to present, amongst various mechanisms, greater total and visceral adiposity [4], impaired glucose tolerance and insulin sensitivity [5,6], higher susceptibility to oxidative stress and inflammation [7], altered metabolic flexibility [8,9,10], mitochondrial dysfunction [11] as well as reduced expression of genes involved in fatty acid metabolism in skeletal muscle [12].

Exercise is a potent intervention to prevent and/or control some, if not most of these alterations and, ultimately, insulin resistance [13,14]. However, the literature offers contrasting evidence regarding the impact of exercise-induced benefits in FDR individuals. While some have observed either no influence of family history [15], or a superior response to exercise in FDR [16], others suggest that FDR could have attenuated [17,18] or delayed [10] benefits from exercise compared to individuals without a family history of T2D. For instance, Irving et al. (2011) showed that while an intensive 9-day training program ameliorated oxidative capacity in both groups, it did not improve insulin sensitivity in the FDR group [18]. In contrast, glucose infusion rate improved by ~50% in individuals without family history of T2D [18]. Allerton et al. (2019) also showed delayed effects of acute high-intensity exercise on metabolic flexibility in young and healthy FDR individuals [10].

The inability to properly select and oxidize substrate offers an attractive mechanistic hypothesis to explain the altered response to exercise in FDR. The capacity to oxidize lipids, especially intramuscular lipids, has been associated with improved insulin sensitivity [19]. Furthermore, a regular turnover of intramuscular lipids limits the accumulation of lipid intermediates such as ceramides and diacylglycerol which disrupt insulin signaling [19]. Hence, some of the metabolic alterations previously mentioned such as altered metabolic flexibility, mitochondrial dysfunction or impaired fatty acid metabolism could potentially explain these hampered exercise-induced benefits in FDR individuals. 

Thus far, metabolic alterations in FDR have been identified in either resting or inactive states. Furthermore, the impact of exercise has been mostly investigated in a chronic setting [15,16,17,18], or after the completion of an exercise session [10]. To the best of our knowledge, the impact of a family history of T2D on substrate metabolism during exercise has not been described. Identifying potential mechanisms that could explain why FDR individuals seem to have impeded exercise-induced benefits could help improve exercise recommendations for this population. The primary objective of this study was thus to assess if a family history of T2D influences exercise metabolism and substrate oxidation during an acute submaximal exercise test in postmenopausal women at risk for T2D. The secondary objectives were to verify if family history of T2D influenced exercise capacity and substrate metabolism and oxidation after acute exercise. 

## 2. Materials and Methods

### 2.1. Participants

In total, 19 inactive women aged between 60 and 75 years old without chronic diseases were included in the study. Participants were divided in two groups based on self-reported family history of T2D which was defined as having a first-degree relative (mother, father, or siblings) diagnosed with the condition. Inclusion criteria were selected to limit factors affecting substrate oxidation [20]. To take part in the study, participants had to be healthy (free of chronic diseases and/or physical incapacity to exercise), be physically inactive (i.e., <75 min of moderate to vigorous planned exercise per week), with a body mass index ≤30 kg/m^2^ [21], be weight stable (±2 kg in the past 6 months), have a normal glycemic control (<6.5% HbA1c and <7 mmol/L fasting glucose), be non-smokers, and not take any medication that could influence lipid and glucose metabolism. 

All participants provided informed written consent before their participation and the study was approved by the Research Ethics Committee of the CIUSSS de l’Estrie-CHUS (#2019-2786).

### 2.2. Protocol Overview

**Figure 1:**
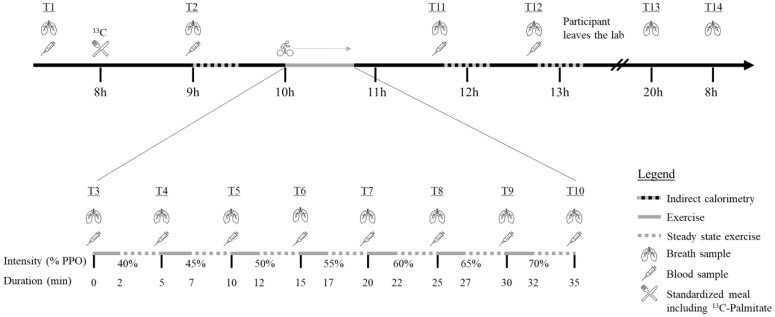
Experimental day % PPO = Percentage of Peak Power Output; min = Minutes; 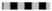 = Indirect calorimetry; 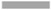 = period of exercise; 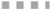 = steady state exercise; 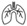 = breath sample; 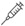 = blood sample; 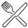 = Standardized meal including ^13^C-Palmitate.

A brief overview of the protocol is first provided, and an in-depth explanation of the methods follows. Briefly, after a phone screening, participants were conveyed to the laboratory of the Research Centre on Aging on three occasions. In a first visit, participants underwent different screening tests to ensure they met the above-mentioned inclusion/exclusion criteria: filled questionnaires regarding medical history and physical activity levels (Physical Activity Scale for the Elderly), anthropometrics and a blood draw. Participants also underwent tests to measure resting metabolic rate, body composition, vital signs, and maximal aerobic capacity. One week later, participants returned to the laboratory for experimental measures (experimental day is detailed in Figure 1). A controlled diet tailored to the participants’ energy requirement was consumed in the 24 h prior to the experimental visit and a standardized breakfast, including a palmitate tracer, was provided on the morning of the visit. Breath and blood samples were collected throughout the day as illustrated in Figure 1. The participants underwent a submaximal exercise test during which breath and blood samples were also collected. Indirect calorimetry was used to measure RER three times throughout the day (once in the morning after breakfast, and twice after exercise). Finally, participants came back to the laboratory 24 h after leaving the previous day with breath samples they self-collected at home, and they were interviewed regarding their nutritional intake during the past 24 h. 

### 2.3. Anthropometrics and Body Composition

Body weight (kg) was measured with an electronic scale (±0.2 kg, SECA707, Hamburg, Germany), height (cm) was measured with a wall stadiometer (Takei, Tokyo, Japan) and BMI was determined as kg/m^2^. Waist circumference was measured to the nearest 0.1 cm using a tape measure 1 cm above the ilium crest. Body composition was measured with dual-energy X-ray absorptiometry (Lunar iDXA, GE Healthcare,Chicago, IL, USA). Total lean body mass (LBM; kg), total fat mass (FM; kg) and fat mass percentage (FM%) were determined in a supine position with a full body scan. The one-week test re-test coefficient of variation in our laboratory for FM is 1.9% and 1.2% for LBM. 

### 2.4. Indirect Calorimetry

Resting metabolic rate (RMR) and resting respiratory exchange ratio (RER) were determined using indirect calorimetry over a 30-min period (CCM express, MGC Diagnostic, Saint-Paul, MN, USA). During the test, participants were laying down in a bed in a dimly lit room with temperatures ranging between 22 and 24 °C. Participants were asked to remain silent, still, and yet awake. The RMR was determined as the average rate of energy expenditure from minutes 11 to 25, inclusively [22]. The first 10 min were removed from the analyses to allow the participant to fully be at rest and last 5 min to avoid the anticipation of the end of the test. To further limit this anticipation, participants had to remove their watches. The intra-class correlation test–retest reliability for the RMR in our facility is 0.98 [0.933; 0.994] (n = 12) at one week. 

The same 30-min protocol was used during the experimental day for the three indirect calorimetry tests to assess metabolic flexibility following a meal (T2) or following acute exercise (T11 and T12).

### 2.5. Diet

Diet was controlled in the 24 h prior to the second visit (submaximal exercise testing). It was tailored to participants energy requirement (based on RMR calculation) with a multiplying factor of 1.4 to account for activities of daily living [23]. The diet had a standard macronutrient distribution: 50% carbohydrate, 35% fat and 15% protein. The menu included whole-wheat bread, peanut butter, commercially available frozen meals, cheese, yogurt, dried fruits, fruit salads and granola bars.

On the morning of the first and second visits, a standardized, caffeine-free breakfast was offered 2 h prior to exercise. The breakfast was comprised of two toasts with peanut butter (36 g), an applesauce (104 mL), cheese (30 g) and fruit juice (250 mL). The single oral dose of 50 mg of ^13^C-Palmitate (Methyl ester, 98% purity, Cambridge Isotope Laboratories Inc., Tewksbury, MA, USA) was added to the first bite of applesauce on the morning of the second visit to measure lipid beta-oxidation rate. 

### 2.6. Maximal Aerobic Capacity

Maximal aerobic capacity (V̇O_2 peak_) was measured using an incremental test on a cycle ergometer in a temperature-controlled room (20–22 °C). Before beginning the test, a resting electrocardiogram (ECG) was performed, and a physician gave clearance to undergo the test. The ECG was maintained throughout the test to measure heart rate and to ensure participants could increase exercise intensity safely. The participants began the test at 20 watts and 10 watts were added every minute until volitional exhaustion. Participants had to maintain a cadence of ≥60 revolutions per minute [24]. V̇O_2_ and V̇CO_2_ were measured with a breath-by-breath system (Medisoft, Sorinnes, Belgium) and were used to determine the RER. V̇O_2 peak_ value was determined as the highest 30-s averaged V̇O_2_ recorded. Peak power output (PPO; in Watts) was defined as the power output during the last completed minute of the maximal test. Participants who could not reach an exercise intensity of ≥70 watts were excluded from the study, due to an inability to properly assign exercise intensities during the experimental day.

### 2.7. Submaximal Exercise Testing

A week after testing maximal aerobic capacity, participants underwent an incremental, submaximal aerobic exercise session on the same cycle ergometer and settings to determine substrate metabolism during steady-state exercise. The test took place two hours after ingesting breakfast and the palmitate tracer. Based on previous results in untrained women for maximal fat oxidation [25], the intensity during the test ranged from 40 to 70% of PPO. The intensity increased by 5% increments for a total of seven stages. Each stage was three minutes long and began after the attainment of a steady state, defined as a variation in heart rate of <3 bpm between two minutes. The average time between two steady state was 2.0 ± 0.4 min and the average duration of those who completed the exercise session was 35.6 ± 2.9 min. Again, a breath-by-breath system was used to measure V̇O_2_ and V̇CO_2_ (Medisoft, Sorinnes, Belgium) and allowed for determination of RER. Heart rate was monitored throughout the test with the Polar H7 heart rate sensor (Polar, Finland) and perceived exertion was also assessed using the 10-point Borg Scale during the second minute of each stage [26]. At the end of each stage, while the participant maintained their pedaling, a blood sample and a breath sample were collected.

### 2.8. Blood Sampling

On the first and second visits, participants arrived fasted, and a blood sample was collected before their breakfast. On the first visit, the blood sample was required to confirm eligibility and fasting glucose and insulin, HbA1c, lipid profile (total cholesterol, HDL-cholesterol, LDL-cholesterol, and triglycerides) and TSH were measured. These analyses were performed by the clinical biochemistry lab of the Centre Hospitalier Universitaire de Sherbrooke using standardized clinic procedures. For the second visit, a catheter was inserted in the antecubital vein of the participant’s forearm and a first fasted blood sample was collected. Then, participants had their breakfast containing the ^13^C-palmitate and a blood sample was collected again one and two hours after the tracer intake. Blood samples were also collected at the end of each exercise stage as well as one and two hours after the end of exercise (Figure 1).

After collection, blood samples were immediately stored on ice for a maximum of 6 h, then centrifuged at 3500 RPM (2851× *g*) for 10 min at 4 °C, aliquoted and stored at −80 °C until analyses. 

### 2.9. Biochemistry

Plasma glucose and lactate (Siemens Healthineers, Erlangen, Germany), non-esterified fatty acids (NEFA) and glycerol (Randox Laboratories, Crumlin, UK) levels were measured with commercially available kits on an automated colorimetric assay (Dimension Xpand plus, Siemens Healthineers, Erlangen, Germany).

Fatty acids (FAs) were extracted from plasma using the Folch et al. method [27] and the details have been previously described [28]. Briefly, 100 µL of a triheptadecanoate solution (TG 17:0, 1.6 mg/mL) was added to 250 µL of plasma as an internal standard. Ten mL of chloroform/methanol (2:1) was then added and the samples incubated for one hour at room temperature. The plasma lipid extract was then saponified using KOH-methanol at 90 °C for one hour, protonated with HCl, and then methylated with BF3-methanol (14%) at 90 °C for 30 min. The ^12^C FAs were then analyzed by gas chromatography with a flame ionization detector (model 6890, Agilent technologies, Santa Clara, CA, USA) as previously described by Chevalier et al. [29].

The measurements of ^13^C palmitate in plasma was performed by gas chromatography-combustion isotope ratio mass spectrometry, as previously described in Goodman & Brenna [30]. The ^13^C:^12^C values immediately before and after exercise, as well as one and two hours after exercise were compared to baseline to determine δ per mil values which were then used to calculate ^13^C-palmitate enrichment in plasma using the equation of Brossard et al. [31]. The determination of plasma palmitate concentrations and ^13^C enrichment in plasma allowed us to account for diverging absorption rates between individuals. 

### 2.10. Breath Sampling

At rest, alveolar breath samples were collected in a vacuumed tube (12 mL exetainer tubes, Labco, UK) using a purpose-built perforated plastic bag attached to a mouthpiece (EasySampler; Quintron Instrument Co., Milwaukee, WI, USA) [32]. Participants exhaled about 500 mL of air before the sample was collected to avoid collecting non-alveolar air. During exercise, to avoid removing the mask and socket of the breath-by-breath system, the mask’s socket was adapted to allow breath sampling. Briefly, an adaptor for the sampling device was added opposite to the gas and volume sampling location of the socket and would be sealed during the steady-state measurements. During breath sampling, the seal was removed, and the sampling device added. Similar to sampling at rest, people would exhale before the sample was collected in vacuumed tubes. Breath samples were all taken in triplicates and pedaling was maintained throughout the sampling procedure. 

Isotopic-ratio mass spectrometry (IRMS; ID Micro Breath CO_2_ analyzer, Compact Science Systems, Newcastle, UK) was used to determine the ratio of ^13^CO_2_:^12^CO_2_ in breath. To determine the Percent Dose Recovery (PDR), we first estimated total blood volume using Nadler’s formula [33]. The percent dose recovered in breath samples was then calculated using the equation from Freemantle et al. (2009) [34], with the exception that indirect calorimetry values were used for V̇CO_2_, instead of the estimation using a constant and body surface area.

### 2.11. Sources of Fat Oxidation

Subcutaneous adipose tissue fatty acids, intramuscular triglycerides, cholesterol (from very-low-density lipoproteins [VLDL]), and dietary lipids are the main fuel sources for lipids oxidation during exercise [20,35]. Given the time between meal and exercise—i.e., two hours—^13^C recovered in breath was considered as an indicator of exogenous/dietary fatty acids oxidation. Plasmatic concentrations of glycerol were considered as representative of adipose tissue lipolysis. It was also shown that the contribution of VLDL-derived triglyceride oxidation to total energy expenditure is relatively negligeable (≤5%) during submaximal aerobic exercise [36]. Therefore, residual fat oxidation was considered to derivate from intramuscular triglyceride sources. 

### 2.12. Statistics

The primary outcome of the study was PDR measured with IRMS. Based on results from previous studies from our group that used ^13^C enrichment and IRMS, we calculated the sample size (α = 0.05, power = 80%) required to show between-group differences in PDR at rest which amounted to 9 per groups. Results are reported as mean ± standard deviation unless otherwise stated. Between group differences in descriptive characteristics were assessed using Mann–Whitney U’s. The Bonferroni method (α/n comparisons) was used to account for multiple comparisons. Group-by-time interactions were calculated with linear mixed models since it uses pairwise deletion rather than listwise deletion as ANOVA tests do. The trapezoidal method was used to calculate area under the curves (AUC) for ^13^C-enrichment in breath and the plasma concentration of ^13^C-palmitate. Aside from AUCs (GraphPad Prism V.9.2, San Diego, CA, USA), all analyses were made using SPSS Statistics V.27 for Windows (IBM, Armonk, NY, USA). Statistical significance was set at *p* ≤ 0.05.

## 3. Results

### 3.1. Population

A total of 25 women was recruited, among whom 19 (10 control and 9 FDR) were included in the analyses. Individuals were excluded from the study for either not meeting the criteria (one was identified with T2D, one had a recent cancer diagnostic, two had abnormal maximal tests and one exercised more than the permitted amount of time) or due to non-compliance with exercise and dietary prescription during the study (n = 1).

Participants were physically inactive, yet healthy, postmenopausal women. Descriptive characteristics are presented in Table 1. The participants were aged 67.6 ± 4.3 years, had a body mass index of 25.9 ± 3.2 kg/m^2^. There were no differences for body composition, resting heart rate and blood pressure, fasting glucose, fasting insulin, HbA1c, lipid profile and resting metabolic rate between the groups (*p ≥* 0.1 *for all*). Exercise capacity (V̇O_2_ peak, PPO and maximal heart rate) was also similar between the two groups (Table 1). Given that PPO was similar between groups, the absolute (W) and relative (W/kg) power outputs during submaximal exercise were also similar between groups (*data not shown*). These similarities allowed between group comparisons without the need for statistical adjustments. 

### 3.2. Primary Outcome: Expired & Plasmatic ^13^C-Palmitate

**Figure 2:**
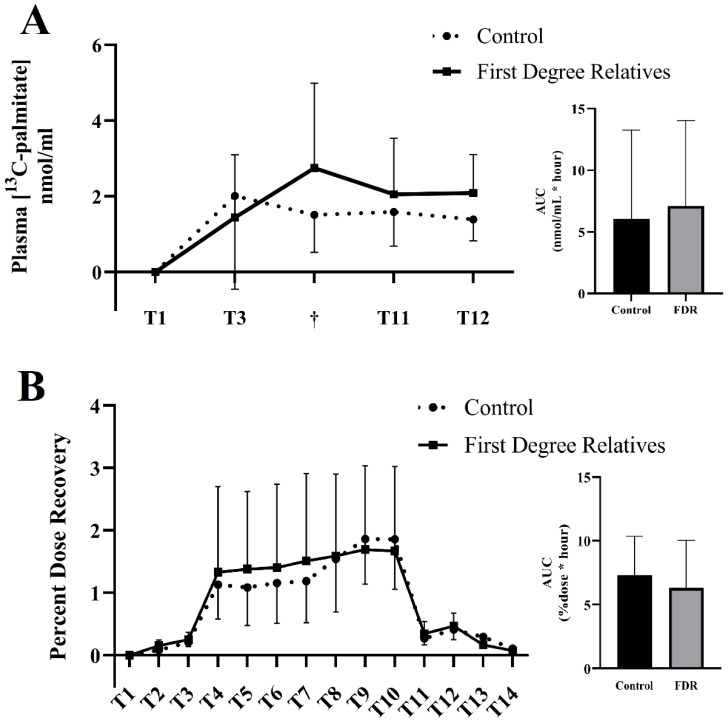
^13^C tracer concentrations in plasma and breath in aging women with or without family history of type 2 diabetes. Concentrations of 13C-palmitate in plasma (**A**) and the percent dose recovery of 13C in breath (**B**) in controls (dotted line) and in first-degree relatives (full line). † Signifies the last stage of exercise completed by the participant (stage 3, n = 2; stage 4, n = 1, stage 5, n = 2; stage 7, n = 14). AUC = Area under the curve. Data shown as mean ± SD.

The ^13^CO_2_ enrichment in breath did not differ between groups at rest, during exercise, in the post exercise period and in the 24 h following ingestion of the tracer (Figure 2; *p =* 0.288). Furthermore, the AUC was similar between groups (Control: 7.35 ± 3.02% recovered × hour; FDR: 6.32 ± 3.71% recovered × hour; *p* = 0.52). 

Plasma palmitate concentrations were similar at baseline before ingestion of the tracer (Control: 74.86 ± 9.54 mg/dL; FDR: 79.07 ± 27.60 mg/dL; *p =* 0.791). There were no between-group differences throughout the experimental day (*p ≥* 0.71 for all).

Plasmatic concentration of ^13^C-palmitate did not differ between groups an hour after ingestion (control: 2.01 ± 2.46 nmol/mL; FDR: 1.97 ± 2.23 nmol/mL; *p =* 0.92), at the end of the exercising session (control: 1.51 ± 0.99 nmol/mL; FDR: 2.97 ± 2.21 nmol/mL; *p* = 0.174) and in the two hours following exercise (control T11: 1.58 ± 0.9 nmol/mL, T12: 1.39 nmol/L ± 0.56 nmol/mL; FDR T11: 2.11 ± 1.39 nmol/mL, T12: 2.1 ± 0.94 nmol/mL (*p* > 0.181 *for both;* Figure 2). The AUC was also similar between the groups (control: 6.07 ± 7.20 nmol/mL × hour; FDR: 7.12 ± 6.93 nmol/mL × hour).

### 3.3. Secondary Outcomes: Blood Metabolites during Submaximal Exercise

Blood metabolites immediately before and during exercise are presented in Table 2. Briefly, lactate and glycerol increased significantly with greater exercise intensity as expected (*p <* 0.001 for both groups), while glucose and NEFA levels remained unchanged (*p ≥* 0.2 for both groups). There were, however, no group differences in any substrate concentrations during submaximal exercise (*p ≥* 0.14 for all). Interestingly, lactate levels showed a group-by-time interaction (*p* = 0.041), while there were no interactions for other metabolites (*p* ≥ 0.28). Plasma concentration of metabolites were also determined one hour and two hours after exercise to evaluate if metabolism differed in the post-exercise period. There were no between-group differences nor group-by-time interactions in plasma concentrations of metabolites in the recovery period (*p* ≥ 0.14 *for all; data not shown*).

**Figure 3:**
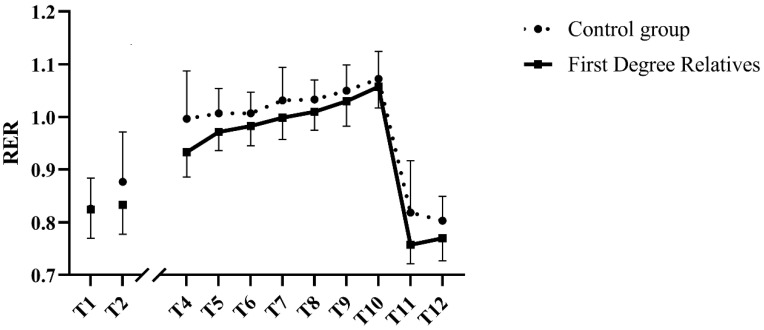
Respiratory exchange ratio before, during and after submaximal exercise in aging women with or without family history of type 2 diabetes. T1 corresponds with resting values taken on the first visit. Data include participants who have not completed the seven stages of exercise. Their data are included up until the last stage of exercise they finished (stage 3, n = 2; stage 4, n = 1, stage 5, n = 2) and resume at T11. AUC = Area under the curve. Data shown as mean ± SD.

RER increased during the exercise session (T4 through T10; *p* = 0.002; Figure 3), without group-by-time interactions (*p* > 0.4 for both). Compared with the last stage of exercise, RER diminished significantly in both groups in the recovery period (*p* < 0.001 *for both*), again without group-by-time interactions (*p* ≥ 0.4 *for both*).

### 3.4. Submaximal Exercise Parameters

In accordance with the incremental nature of the submaximal exercise session, heart-rate reserve, perceived exertion as well as V̇O_2_ and V̇CO_2_ increased with time (*p* < 0.001 for all), without group-by-time interactions (*p* ≥ 0.37 for all; Table 3). 

If the percentage of heart rate reserved increased in both groups during exercise (Table 3), there was however a between-group difference for the percentage of heart-rate reserve with FDR consistently having a lower percentage at each stage (*p* = 0.012).

A total of 14 participants completed the seven stages of the submaximal exercise session, five in the control group (50%) and nine in the FDR group (100%). The five control participants who ended the test prematurely did so on stages 3 (n = 2), 4 (n = 1) and 5 (n = 2) out of 7, which, respectively, represent 50, 55 and 60% of PPO. Lactate levels at the last stage for these participants was, on average, 4.46 mmol/L (range: 3.17–6.44), average heart rate reserve was 82% (range 62 to 100%), and RER was consistently above one, suggesting the attainment of a high intensity.

There were differences between control participants who completed the test and those who did not. Those who did not complete the test were the five lowest ranking in terms of aerobic capacity (completed: 21.1 mlO_2_/kg/min, range: 18.1–28.6 mlO_2_/kg/min; not completed: mean: 16.3 mlO_2_/kg/min, range: 15.8–17.5 mlO_2_/kg/min; *p* = 0.009) and peak power output (completed: mean: 108 W, range: 90–140 W; not completed: mean: 80 W, range: 70–90 W; *p* = 0.023) in the control group. 

## 4. Discussion

We hypothesized that a family history of T2D would alter substrate metabolism and oxidation during submaximal exercise and in the recovery period immediately after exercise in healthy postmenopausal women. Our results do not support our hypothesis and show that family history of T2D does not alter substrate metabolism and oxidation during submaximal exercise in postmenopausal women. This is, to the best of our knowledge, the first study to investigate the impact of family history of T2D on metabolism during acute exercise. 

The main observation from this study is that family history of T2D does not seem to influence substrate oxidation and metabolism during an incremental aerobic exercise nor in the immediate period after exercise in healthy postmenopausal women. Indeed, we showed no between-group differences in plasmatic concentrations of glucose, lactate, NEFA and glycerol, nor in substrate oxidation throughout the study. Our observations are contrasting with previous studies from Russel et al. and Allerton et al. which have reported metabolic inflexibility in healthy young FDR individuals following passive stretching [9] or acute exercise [10]. In both studies, FDR individuals had an inability to suppress fat oxidation following oral glucose tolerance test [9] or a mixed-meal tolerance test [10], leading to an attenuated increase in RER compared with healthy controls. The differences between our results and those of others could reside in the context of the measurements—i.e., the metabolic stress induced. Other studies have investigated metabolic inflexibility in a post-prandial context [8,9,10], or during a euglycemic-hyperinsulinemic clamp [8]. These are all contexts of energy surplus, where the action of insulin is primordial. In contrast, our study focused on exercise metabolism, a context of energy demand during which the role of insulin is minimal [37]. Our results therefore suggest that the metabolic defects previously observed in family history of T2D might be limited to contexts where the action of insulin is preponderant, and that exercise metabolism is spared in a healthy population of FDR. It would however be important to confirm that the observations from this study are replicable in other types of exercise modalities such as continuous aerobic, high intensity interval training or even resistance exercise. 

A second important conclusion from our study is that no differences were observed in the ratio of exogenous to endogenous fat oxidized during submaximal exercise. Furthermore, albeit from indirect measurements, our observations do not support an altered intramuscular lipid turnover during exercise. These results contrast with those of Eriksson et al. who showed attenuated insulin-mediated inhibition of lipolysis [38] and Dahlman et al. who reported greater unstimulated adipose tissue lipolysis in FDR individuals compared with individuals without family history of T2D [39]. Again, it seems the metabolic defects reported in insulin-stimulated conditions [38] or at rest [39] do not transpose to exercise. 

It should be taken into consideration that most of the previous studies have studied the impact of family history of T2D in young individuals with insulin resistance while the population studied in this research are healthy postmenopausal women. It is therefore possible that this population is either protected from the metabolic alterations observed in younger population, or the increased risk for T2D associated with the menopausal transition masks the differences imposed by family history of T2D. 

An unexpected observation from our study is that while every FDR participant completed the submaximal exercise test, half the control participants were unable to complete the test. It is also interesting to note that FDR participants were exercising at a lower percentage of their HRR throughout the submaximal exercise test, which is in line with the modest, yet statistically significant slower increase in plasma lactate concentration with exercise intensity in the FDR group. The control participants who did not complete the test had the lowest V̇O_2_ peak and PPO, yet the FDR participants with similar exercise capacity were able to complete the incremental test. These results could not be explained by differences in maximal aerobic capacity, or peak power output during V̇O_2_ max. Furthermore, exercise was tailored to each participant based on their maximal test and the relative effort (percentage of PPO) was similar between groups. Others have reported surprising results to physical tests in young, healthy FDR athletes [40]. In their study, Bianco et al. (2014) showed that FDR athletes displayed greater squat jump height and Wingate results compared to controls without family history of T2D [40]. A greater proportion of type IIb muscle fibers in FDR, as shown by Nyholm et al. (1997), could potentially explain our observations [41]. These results suggest greater explosive power in FDR population, which could have played a role in the context of the cycling test we used. Finally, between-group differences in ventilatory thresholds could potentially explain the differences we observed and should be further investigated.

This study had strength and limitations. The usage of a highly purified and uniformly labelled tracer allowed us to determine the contribution of exogenous lipid to fat oxidation during exercise. Furthermore, exercise prescription was tailored to the participants capacity based on a maximal test and not through predicting equations. The homogenous sample limits the ability to generalize data to other populations and are only applicable to healthy postmenopausal women. The contribution of intramuscular lipid to fat oxidation was determined through indirect measurements and more direct approaches are still required to confirm our results. Finally, the sample size calculations were based on the primary outcome and other analyses could be underpowered. It would therefore be important for studies to confirm our results with an adequate sample size. 

## 5. Conclusions

Altogether, our results suggest that substrate metabolism and oxidation during submaximal exercise is not altered in healthy postmenopausal women with a family history of T2D. Based on our result, the alteration of substrate oxidation and metabolism during exercise is therefore not a participating mechanism to the delayed responses to exercise-induced benefits observed in family history of T2D. More studies are thus required to identify the metabolic alterations contributing to delayed and hampered exercise-induced benefits and if age may affect this process.

## Figures and Tables

**Table 1:** Descriptive characteristics.

	Control (n = 10)	FDR (n = 9)
Age (years)	67.7 ± 4.7	67.6 ± 4.2
Height (m)	1.60 ± 0.07	1.58 ± 0.06
Weight (kg)	65.3 ± 6.4	65.6 ± 9.9
BMI (kg/m^2^)	25.6 ± 2.2	26.3 ± 4.2
Waist Circumference (cm)	101.9 ± 20.8	97.5 ± 8.8
Fat-free mass (kg)	36.4 ± 3.2	38.2 ± 4.6
Fat mass (kg)	26.4 ± 5.5	25.5 ± 5.4
Fat mass (%)	41.8 ± 5.5	39.8 ± 4.7
*Resting vital signs & metabolism*		
Resting Heart Rate (bpm)	62.4 ± 8.0	62.89 ± 2.62
Systolic blood pressure (mmHg)	121.5 ± 6.6	122.7 ± 13.9
Diastolic blood pressure (mmHg)	75.0 ± 6.0	73.7 ± 7.6
Resting metabolic rate (kcal/day)	1099.5 ± 120.4	1151.1 ± 248.3
*Fasting blood samples*		
Fasting glucose (mmol/L)	4.68 ± 0.46	4.57 ± 0.49
Fasting insulin (pmol/L)	36.67 ± 8.96	67.13 ± 43.30
HbA1c (%)	5.65 ± 0.26	5.47 ± 0.23
HDL-cholesterol (mmol/L)	1.94 ± 0.49	1.62 ± 0.42
LDL-cholesterol (mmol/L)	3.55 ± 1.02	3.63 ± 0.78
Total Cholesterol (mmol/L)	6.00 ± 1.06	5.82 ± 1.06
Triglycerides (mmol/L)	1.13 ± 0.46	1.26 ± 0.60
*Maximal aerobic capacity*		
Absolute V̇O_2_ peak (L/min)	1.2 ± 0.3	1.3 ± 0.2
Relative V̇O_2_ peak (ml/kg/min)	18.7 ± 3.8	20.4 ± 2.7
Peak Power Output (W)	94.0 ± 21.7	95.6 ± 15.9
Peak Heart Rate (bpm)	153.6 ± 15.6	149.9 ± 9.5

BMI: Body Mass Index; HDL: High density lipids; LDL: Low density lipids; Comparison with bilateral U’s of Mann–Whitney tests: *p* > 0.1 for all.

**Table 2:** Plasma metabolites immediately before and during submaximal exercise.

		Pre-Exercise	Stage 1	Stage 2	Stage 3	Stage 4	Stage 5	Stage 6	Stage 7
Glucose (mmol/L)	Control	5.19 ± 1.02	5.42 ± 0.86	5.46 ± 0.68	5.36 ± 0.67	5.31 ± 0.61	5.01 ± 0.43	5.49 ± 0.92	5.26 ± 0.51
	FDR	5.71 ± 0.81	5.89 ± 1.02	6.00 ± 0.92	5.44 ± 0.75	5.46 ± 0.96	5.54 ± 0.92	5.41 ± 1.01	5.68 ± 0.81
Lactate (mmol/L) ^a,b^	Control	1.30 ± 0.29	2.29 ± 0.68	3.02 ± 1.20	3.43 ± 0.94	4.01 ± 1.15	4.53 ± 1.21	5.70 ± 1.51	6.49 ± 1.30
	FDR	1.55 ± 0.69	2.11 ± 0.57	2.86 ± 0.88	3.05 ± 0.73	3.66 ± 1.39	4.30 ± 1.59	4.92 ± 1.62	5.91 ± 2.18
NEFA (mmol/L)	Control	0.37 ± 0.14	0.36 ± 0.17	0.41 ± 0.24	0.39 ± 0.19	0.31 ± 0.07	0.37 ± 0.14	0.36 ± 0.09	0.38 ± 0.08
	FDR	0.40 ± 0.22	0.47 ± 0.26	0.35 ± 0.19	0.4 ± 0.27	0.41 ± 0.18	0.40 ± 0.18	0.47 ± 0.24	0.46 ± 0.21
Glycerol (μmol/L) ^a^	Control	80.31 ± 30.48	77.14 ± 20.73	90.81 ± 29.13	112.56 ± 40.70	98.65 ± 13.25	-	154.66 ± 28.57	157.22 ± 13.95
	FDR	89.14 ± 46.53	99.12 ± 53.79	97.11 ± 43.24	99.61 ± 56.68	111.25 ± 58.32	107.57 ± 42.65	152.62 ± 78.65	171.19 ± 71.99

FDR: First-Degree Relatives; NEFA: Non-Esterified Fatty Acids; Data are presented as mean ± SD. ^a^ Main effect of time, *p* < 0.001; ^b^ Time by group interaction, *p* < 0.05

**Table 3:** Submaximal exercise parameters.

		Stage 1	Stage 2	Stage 3	Stage 4	Stage 5	Stage 6	Stage 7
Heart Rate Reserve (%) ^a,b^	Control	56.3 ± 12.4	64.0 ± 12.7	70.9 ± 12.0	78.9 ± 12.3	85.8 ± 12.0	89.2 ± 8.0	95.0 ± 6.5
	FDR	41.5 ± 8.7	48.8 ± 8.6	55.9 ± 10.0	64.7 ± 10.4	70.6 ± 10.3	78.0 ± 9.3	83.2 ± 9.9
Perceived exertion ^a^	Control	2.0 ± 1.0	2.5 ± 1.3	3.3 ± 1.7	3.2 ± 1.3	3.9 ± 1.1	3.9 ± 0.7	4.8 ± 1.1
	FDR	1.4 ± 0.8	2.0 ± 1.4	2.1 ± 0.9	2.7 ± 0.8	3.3 ± 1.2	3.9 ± 1.2	4.3 ± 1.1
V̇O_2_ (L/min) ^a^	Control	0.88 ± 0.13	0.9 ± 0.2	0.93 ± 0.23	1.02 ± 0.28	1.14 ± 0.31	1.2 ± 0.35	1.36 ± 0.34
	FDR	0.89 ± 0.13	0.91 ± 0.14	0.95 ± 0.12	1.01 ± 0.12	1.05 ± 0.15	1.1 ± 0.17	1.13 ± 0.16
V̇CO_2_ (L/min) ^a^	Control	0.87 ± 0.09	0.90 ± 0.17	0.93 ± 0.21	1.05 ± 0.26	1.17 ± 0.28	1.35 ± 0.26	1.41 ± 0.26
	FDR	0.83 ± 0.14	0.88 ± 0.15	0.93 ± 0.14	1.01 ± 0.13	1.06 ± 0.16	1.13 ± 0.18	1.20 ± 0.18

FDR: First-Degree Relatives; V̇O_2_: Rate of oxygen consumed; V̇CO_2_: Rate of carbon dioxide expired. ^a^ Main effect of time, *p* < 0.001; ^b^ Main effect of group, *p* = 0.012.

## Data Availability

The data presented in this study are available on request from the corresponding author.
